# Screening for Hypertension in Children and Adolescents: Methodology and Current Practice Recommendations

**DOI:** 10.3389/fped.2017.00051

**Published:** 2017-03-15

**Authors:** Michaela N. Lewis, Ibrahim F. Shatat, Shannon M. Phillips

**Affiliations:** ^1^Medical University of South Carolina, Charleston, SC, USA

**Keywords:** blood pressure measurement, hypertension, ambulatory monitoring, children, oscillometric

## Abstract

Hypertension (HTN) requires urgent, uniform, and consistent attention across all frontiers of pediatric health care not only because of established links between the onset of HTN during one’s youth and its sustenance throughout adulthood but also because of the sequelae associated with the disease’s trajectory, such as cardiovascular disease, end organ damage, and decreased quality of life. Although national guidelines for the diagnosis and management of pediatric HTN have been available for nearly 40 years, knowledge and recognition of the problem by clinicians remain poor due to a host of influencing factors. The purpose of this article is to explicate key issues contributing to the inaccurate measurement of blood pressure and misclassification of HTN among children and to present strategies to address these issues.

## Introduction

Hypertension (HTN) requires urgent, uniform, and consistent attention across all frontiers of pediatric health care not only because of established links between the onset of HTN during one’s youth and its sustenance throughout adulthood but also because of the sequelae associated with the disease’s trajectory, such as cardiovascular disease (CVD), end organ damage, and decreased quality of life.

Between 2011 and 2014, the prevalence of HTN among children and adolescents aged 8–17 years was approximately 2.2% (1), a number similar to other chronic childhood illnesses that garner more attention, such as asthma (9%), autism (1%), or epilepsy (1%) (2). Up to 30% of newly diagnosed hypertensive children demonstrate significant target organ damage, particularly left ventricular hypertrophy (2). Pediatric HTN is an issue attracting attention on a national level, as the reduction of the proportion of children with HTN is an active objective of the Healthy People 2020 movement (1).

Although national guidelines for the diagnosis and management of pediatric HTN have been available for nearly 40 years, knowledge and recognition of the problem by clinicians remain poor due to a host of influencing factors (3). Faulty blood pressure (BP) testing can lead to misdiagnosis and unnecessary treatment, so proper BP measurement methods are essential. To obtain accurate BP measurements, clinicians must consider the type of equipment to be used, training personnel on proper technique, the environment in which measurement is conducted, classification of BP according to recommendations, and assessment for factors that could affect BP. The purpose of this paper is to provide an overview of practice recommendations, including a discussion of factors that may impact a provider’s ability to appropriately assess pediatric patients for HTN.

## Practice Recommendations

The United States Preventive Services Task Force states that the current evidence is insufficient to assess the balance of benefits and harms of screening for primary HTN in asymptomatic children and adolescents to prevent subsequent CVD in childhood or adulthood (4). The lack of compelling evidence to universally screen healthy children was also highlighted in a review by Chiolero and colleagues (5). However, given the implications of HTN and CVD in adulthood, a lifespan approach of early screening has been advocated by the National Heart, Lung, and Blood Institute (NHLBI) (6) and the American Heart Association (AHA) (7).

The AHA (7), NHLBI (6, 8), and American Association of Pediatrics (9) recommend that children over 3 years and under 18 years receive a BP measurement at least annually, and once during every health-care episode. Children under 3 years should have their BP measured in special circumstances such as a history of being very low birth weight, history of recurrent urinary tract infections, and congenital heart disease.

### Device Selection

When selecting a device, clinicians must consider the necessary equipment, advantages, and disadvantages associated with each option. The most commonly used devices are the mercury sphygmomanometer, the aneroid sphygmomanometer, the office oscillometric device (single and serial measure), and the ambulatory blood pressure monitor (ABPM). Table 1 outlines key considerations for each device.

**Table 1:** **Blood pressure measurement device selection**.

Device	Equipment	Advantages	Disadvantages and other considerations
Mercury sphygmomanometer	Mercury column Stethoscope Appropriately sized cuff with bladder and inflation bulb	Considered the “gold standard” Directly comparable to classification tables normative data	Environmental concerns about mercury contamination Potential for error and bias
Aneroid sphygmomanometer	Lever and bellow aneroid unit Stethoscope Appropriately sized cuff with bladder and inflation bulb	Ecologically friendly alternative to mercury sphygmomanometer	Tends to be less accurate than mercury Susceptible to reading errors resulting from trauma to the unit Requires regular calibration Potential for error and bias
Office oscillometric (single measure)	Automated unit validated in pediatric populations Appropriately sized cuff	Easier to use than sphygmomanometer Ecologically friendly Less susceptible to error and bias Easier to use in situations when auscultation is challenging Beneficial in situations when frequent measurement is necessary Cuff placement less critical	Wide variation in devices Device should be validated in children prior to use Susceptible to inaccurate readings in certain situations Susceptible to movement artifact
Office oscillometric (serial measure)	Automated unit validated in pediatric populations Appropriately sized cuff	Can assist with accurate diagnosis of hypertension (HTN) Reduces effect of white-coat HTN and masked HTN Cost-effective alternative to ambulatory blood pressure monitor	Few units validated in pediatric populations
Ambulatory	Automated unit for home use validated in pediatric populations Unit able to store and download data Appropriately sized cuff	Can assist with accurate diagnosis of HTN Reduces effect of white-coat HTN and masked HTN Can identify non-dipping BP pattern Can monitor BP patterns over time	May not be well tolerated in children Requires family training and cooperation Susceptible to misreporting Normative values differ by sex and race Requires consultation to pediatric HTN specialist Equipment and analysis can be costly

The gold standard is auscultation using a mercury sphygmomanometer; NHLBI BP classification tables are based on auscultatory measurements (6, 7). Since use of an appropriately sized cuff is critical for accurate measurement, the AHA advises clinicians have the following cuff sizes on hand: newborn/premature infant (4 cm × 8 cm), infant (6 cm × 12 cm), older child (9 cm × 18 cm), standard adult, large adult, and thigh for leg measurement or for children with large arms (7). The bladder width should be at least 40% of the patient’s arm circumference midway between the olecranon and acromion processes and should cover 80–100% of the circumference of the arm (6, 7).

While the mercury sphygmomanometer is considered the gold standard, concerns have arisen about mercury contamination leading to this device being banned in some locations (7). The aneroid sphygmomanometer is a more environmentally friendly alternative that uses metal bellows and levers to register pressure. However, the aneroid sphygmomanometer tends to be less accurate than the mercury sphygmomanometer and requires regular calibration. Wall-mounted aneroid units are less likely to be dropped or subject to trauma and are more accurate than mobile devices (10).

To measure BP using either the mercury or aneroid sphygmomanometer, the clinician inflates a cuff to a pressure greater than the SBP, thus occluding the brachial artery, then gradually deflates the cuff to auscultate sounds using a stethoscope placed at the artery below the cuff. A disadvantage of both options is the potential for observer error, such as differences in training, terminal digit preference, and expectation bias due to knowledge of previous readings (5).

Oscillometric (automated) BP measurement devices have rapidly replaced sphygmomanometers in clinical practice. These devices are more ecologically friendly, easier to use, and eliminate potential sources of bias. Oscillometric devices are also beneficial in situations in which auscultation is challenging, such as with newborns and young infants, where device utilization requires tolerance of excessive motion, and in intensive care settings when repeated measurements are required (7, 11). However, there is wide variation in devices, arterial stiffness and wide pulse pressure can lead to inaccurate readings, and measurement is susceptible to movement artifact.

When selecting an oscillometric device, clinicians should seek instruments that have been validated in the pediatric population (6, 12). The Association for the Advancement of Medical Instrumentation and the British Hypertension Society each developed established protocols for validation, and more recently, the European Society of Hypertension Working Group on Blood Pressure Monitoring developed an International Protocol to validate BP measurement devices (7, 13). To assist clinicians in identifying devices that have been validated and recommended, the dabl Educational established a not-for-profit, foundation-funded website to provide up-to-date validation information on BP measurement devices^1^ (14). Clinicians may consult these tables to determine which devices are recommended for use in children.

Serial-measure oscillometric devices are designed to obtain a series of BP measurements at intervals of 1 min or more with the patient seated alone in a quiet room in the office setting. The number of serial measurements varies by device, typically three to six, and an average is calculated. In adults, no significant difference has been found between BP classification using serial measurement technique versus 24-h ABPM. The serial measure oscillometric device, such as the BpTru, can substantially reduce the effect of white coat HTN and masked HTN and can be a cost-efficient alternative to ABPM (15–17). The BpTru has been validated in children aged 3–18 years (18).

In some cases, ABPM is warranted to obtain a more accurate representation of the BP. With this method, BP is measured repeatedly, usually over a 24-h period, and the measurements are stored and downloaded (6, 7). ABPM is indicated when white coat HTN is suspected, to identify a non-dipping pressure pattern, to monitor drug effects (6, 7), and in situations when it is necessary to monitor BP patterns, such as chronic kidney disease, autonomic dysfunction (6), target-organ damage, and low birth weight (12).

Various ABPMs are available, but few have been formally validated in children (11). Beyond ensuring validation of a device, clinicians should identify a monitor that is lightweight, able to tolerate movement, and has pediatric cuffs. Software should be programmable to measure every 15–20 min over 24-h period, and preferably, can provide a report with pediatric reference data (19). ABPM can be conducted with a wide age range of children (20), but may not be well tolerated in all children, requires family training and cooperation, is susceptible to misreporting (12), and equipment and monitoring procedures are costly. Further, normative values differ by sex, height, and race (21, 22).

### Care of Equipment

Equipment must be properly maintained to ensure accurate measurement. Hardware should be cleaned with disinfecting wipes, and ABPM cuff covers should be washed between patients (11). The column of mercury sphygmomanometers should be clean, should rise and fall freely, and the upper curve of the meniscus should rest at 0 mmHg. Aneroid and other non-mercury sphygmomanometers should be calibrated semiannually by connecting the manometer to mercury column or electronic testing device. The needle should rest at 0 mmHg and should read within 4 mmHg of the mercury sphygmomanometer when inflated to 100 and 200 mmHg (6, 7). Since various automated devices are available on the market, calibration should occur according to the manufacturer’s recommendations (11).

### Performance Considerations

The use of proper technique when obtaining BP readings is vital in terms of obtaining an accurate measurement and essential in reducing procedure variability. When possible, the patient should be seated with the antecubital fossa supported at heart level. The patient’s feet should be on the floor, not dangling from an exam table. Clinicians should remove clothing that covers the location of the cuff. The patient’s legs should be uncrossed, with the back and arm supported (7). A cuff placed over clothing can cause a 5–50 mmHg discrepancy in SBP, an unsupported back can cause a 6–10 mmHg discrepancy in SBP, and sitting with the arm unsupported can cause a 1–7 mmHg in SBP and a 5–11 mmHg discrepancy in DBP (23).

In addition to proper positioning, the patient should be in a quiet environment. The clinician should avoid overinflating the cuff, since an overly tight cuff can cause upset in young children. Avoid talking during measurement (7), since talking and active listening can cause a 10-mmHg discrepancy in SBP and DBP (23).

Using the appropriate cuff size is another critical factor, since a too-small cuff can lead to false high readings and a too-large cuff can lead to false low readings (6). A too-small cuff can cause a discrepancy of 10 mmHg in SBP and 2–8 mmHg in DBP (23). Standard cuff placement is the upper arm; the right arm is the preferred site for repeated measures to maintain consistency and for accurate comparison to standards (6). In some circumstances, such as arm fracture or in the case of acute illness, it may be necessary to measure in an alternate site or position. It is important to note that pressures vary by location and position.

### BP Classification

Accurate BP classification requires assessment of the patient’s age, sex, and height (7). The clinician first determines the patient’s height percentile using Centers for Disease Control and Prevention growth charts, then the clinician refers to the NHLBI (6) standardized BP classification tables to categorize the BP as normotensive, prehypertensive, or hypertensive. The AHA and the NHLBI recommend the patient’s BP be measured and recorded at least twice at each occasion, and the average of SBP and DBP be recorded (6, 7). However, Becton and colleagues (24) conducted an evaluation of NHANES data and found that in greater than 90% of adolescents aged 13–18 years, the BP classification remained the same in repeat sequential measurements.

Once the BP is plotted on the appropriate table for age, sex, and height, the percentile ranking is assessed. A BP that falls below the 90th percentile is considered normotensive, while a BP that falls between the 90th and 95th percentile is classified as prehypertension. Further, adolescents with a BP greater than 120/80 should also be considered prehypertensive. Patients with a BP that falls into the prehypertensive range should be reassessed and evaluated for other risk factors (6). A BP that, on repeated assessments, consistently falls equal to or greater than the 95th percentile for age, sex, and height is classified as hypertensive (6, 7). If a BP falls between the 95th percentile and the 99th percentile plus 5 mmHg, the measurement should be repeated on two additional occasions to confirm the classification. However, if the BP is greater than the 99th percentile plus 5 mmHg, the patient should be promptly referred to a specialist for evaluation (6).

Before diagnosing a patient as hypertensive, the elevated BP should be confirmed on repeat visits, as BP measurement is more accurate if averaged over weeks or months rather than at a single visit (6, 7). An exception for this recommendation is when a patient appears symptomatic or has a profoundly elevated BP, in which case the patient should be promptly referred for further evaluation.

Pediatric HTN experts have developed and validated quick reference tables that are easier to use in the busy clinic setting than the complex NHLBI standardized BP classification tables. One example is “A Pocket Guide to Blood Pressure Measurement in Children” developed by the National High Blood Pressure Education Program Working Group on High Blood Pressure in Children and Adolescents (25). This reference guide is published online by the NHLBI^2^ and offers tips on measurement, classification, and interpretation, and a simplified table by age and height.

### Other Special Considerations

Clinicians must also assess for factors that could affect BP, such as routine or intermittent medication use, recent use of tobacco products, and consumption of a sodium-rich or high caffeine diet. Multiple over-the-counter, herbal, and prescription medications have the potential to elevate BP, including decongestants, ephedra, ginkgo, ginseng, senna, St. John’s wort, and oral contraceptives. Clinicians should also consider whether the patient is being treated for ADHD, since some medications prescribed for this condition may elevate BP. It is also important to assess for use of illegal substances such as anabolic steroids, amphetamines, and cocaine, all of which can affect BP (26).

Use of tobacco products can cause masked HTN (7), and smoking within 30 min of BP measurement can cause a 6–20 mmHg discrepancy in SBP (23). Clinicians should ask questions directed at determining recent tobacco use, but should also inquire about sustained tobacco use since this can cause arterial changes that may affect BP. Smoking in the home, by parents or other residents, should also be assessed, because secondhand smoke can increase BP (27). Clinicians should also consider e-cigarette use, which can increase the DBP; e-cigarette use tripled among middle and high school students from 2013 to 2014 (28).

Finally, clinicians should consider the effect of dietary habits and sleep habits when assessing BP. Increased consumption of processed foods and those high in sodium, and poor sleep are associated with an elevated SBP (12), and caffeine use has been associated with masked HTN (7).

## Conclusion

Given the implications of CVD in adulthood, a lifespan approach of early screening for HTN in children is essential in reducing the mortality and morbidity associated with vascular compromise. While this article provides an overview of practice recommendations, current guidelines are actively being revised and updated. Adherence to recommended pediatric HTN guidelines through the use of accurate screening measures and practices and remaining abreast of practice guideline updates is the first step down the path of healing.

## Author Contributions

All persons who meet authorship criteria are listed as authors, and all authors certify that they have participated sufficiently in the work to take public responsibility for the content, including participation in the concept, design, analysis, writing, or revision of the manuscript.

## Conflict of Interest Statement

The authors declare that the manuscript was written in the absence of any commercial or financial relationships that could be construed as a potential conflict of interest.

## Footnotes

^1^http://www.dableducational.org/welcome.html.

^2^https://www.nhlbi.nih.gov/files/docs/bp_child_pocket.pdf.
